# PMG-SAM: Boosting Auto-Segmentation of SAM with Pre-Mask Guidance

**DOI:** 10.3390/s26020365

**Published:** 2026-01-06

**Authors:** Jixue Gao, Xiaoyan Jiang, Anjie Wang, Yongbin Gao, Zhijun Fang, Michael S. Lew

**Affiliations:** 1 School of Electronic and Electrical Engineering, Shanghai University of Engineering Science, Shanghai 201620, China; m320123419@sues.edu.cn (J.G.); gaoyongbin@sues.edu.cn (Y.G.); 2 School of Electronic and Computer Engineering, Peking University, Beijing 100871, China; ajwang@stu.pku.edu.cn; 3 School of Computer Science and Technology, Donghua University, Shanghai 201620, China; zjfang@dhu.edu.cn; 4 Leiden Institute of Advanced Computer Science, Leiden University, EZ 2311 Leiden, The Netherlands; m.s.lew@liacs.leidenuniv.nl

**Keywords:** SAM, image segmentation, pre-mask guidance, automatic segmentation, dichotomous image segmentation

## Abstract

The Segment Anything Model (SAM), a foundational vision model, struggles with fully automatic segmentation of specific objects. Its “segment everything” mode, reliant on a grid-based prompt strategy, suffers from localization blindness and computational redundancy, leading to poor performance on tasks like Dichotomous Image Segmentation (DIS). To address this, we propose PMG-SAM, a framework that introduces a Pre-Mask Guided paradigm for automatic targeted segmentation. Our method employs a dual-branch encoder to generate a coarse global Pre-Mask, which then acts as a dense internal prompt to guide the segmentation decoder. A key component, our proposed Dense Residual Fusion Module (DRFM), iteratively co-refines multi-scale features to significantly enhance the Pre-Mask’s quality. Extensive experiments on challenging DIS and Camouflaged Object Segmentation (COS) tasks validate our approach. On the DIS-TE2 benchmark, PMG-SAM boosts the maximal F-measure from SAM’s 0.283 to 0.815. Notably, our fully automatic model’s performance surpasses even the ground-truth bounding box prompted modes of SAM and SAM2, while using only 22.9 M trainable parameters (58.8% of SAM2-Tiny). PMG-SAM thus presents an efficient and accurate paradigm for resolving the localization bottleneck of large vision models in prompt-free scenarios.

## 1. Introduction

The paradigm of building large-scale foundation models, first established in Natural Language Processing (NLP), has now firmly taken root in Computer Vision, aiming to create single, powerful models capable of addressing a wide array of visual tasks. Among the myriad of visual tasks, image segmentation, which involves fine-grained, pixel-level understanding, stands as a cornerstone for in-depth scene perception. A class-agnostic segmentation model capable of segmenting any object in any image is not only a long-sought goal in academia but also a core technology driving critical applications such as autonomous driving and medical image analysis. The advent of the Segment Anything Model (SAM) [1] in 2023, with its remarkable zero-shot generalization capabilities, has propelled the concept of a class-agnostic segmentation model to new heights.

**Figure 1:**
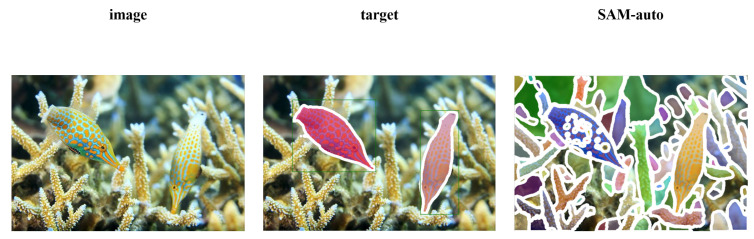
Examples of fragmented segmentation and semantic incoherence in automatic mode of SAM.

The remarkable power of SAM stems from its interactive, prompt-based design, allowing it to segment virtually any object specified by user-provided cues such as points, boxes, or masks. However, a significant gap exists between its “segment everything” automatic mode and the needs of many real-world applications that require fully automatic segmentation of specific objects of interest. The automatic mode of SAM employs an exhaustive strategy, placing a dense grid of points across the image without any localization guidance. While enabling prompt-free operation, this design philosophy leads to two critical deficiencies: first, localization blindness, where the model fails to identify and prioritize salient objects, resulting in fragmented and semantically incoherent masks (Figure 1), and second, computational redundancy, expending resources on irrelevant background regions. Consequently, its performance on targeted segmentation tasks like Dichotomous Image Segmentation (DIS) [2] is severely compromised. As research [3,4] shows, the automatic mode’s performance is drastically inferior to its prompt-guided counterpart, even when the latter is provided with a simple ground-truth bounding box (GT-Bbox). This highlights a core bottleneck: SAM’s original automatic design lacks the intrinsic capability for targeted, autonomous localization.

To overcome these limitations, existing research has predominantly explored two directions: enhancing precision through more sophisticated prompt engineering or user interaction [5,6], or adapting the model to specific domains via adapters or fine-tuning [7,8]. The former, however, compromises the autonomy of model, while the latter risks impairing its valuable generalization ability.

Diverging from these approaches, our work addresses the problem at a more fundamental level. Instead of generating sparse external prompts or performing post hoc refinement, we propose to redesign the information flow within the automatic segmentation process itself. We introduce an internal, end-to-end guidance mechanism that operates without manual prompts. This mechanism first performs a coarse global analysis to efficiently generate a preliminary mask identifying salient regions, and then leverages this mask to guide a fine, detailed segmentation within those specific areas.

To this end, we propose a Pre-Mask Guided framework for SAM (PMG-SAM). The central concept is inspired by human cognitive processes: first, a lightweight, dual-branch encoder rapidly generates a global Pre-Mask that identifies potential objects of interest within the image. Subsequently, this Pre-Mask is utilized as a high-quality internal prompt to guide a powerful decoder in performing fine-grained contour segmentation. This coarse-to-fine mechanism replaces the exhaustive search with globally informed guidance, addressing the position sensitivity issue at its source and opening a new avenue for designing efficient, class-agnostic segmentation models. In summary, the contributions of this paper are fourfold as follows:

- We propose a “locate-and-refine” paradigm implemented through Pre-Mask Guidance. This approach shifts SAM’s automatic mode from an exhaustive grid-based search to a targeted process, directly addressing the core issue of localization blindness by providing a dense, global prior before segmentation.
- We present an effective implementation of this paradigm, featuring a dual-branch encoder that leverages the complementary strengths of Transformer and CNN architectures. A novel Dense Residual Fusion Module (DRFM) is designed to synergistically fuse these features, generating a high-quality Pre-Mask that is crucial for the guidance mechanism.
- To enhance boundary details in the “refine” stage, we integrate a high-resolution feature injection pathway. This pathway preserves crucial spatial information from the encoder, leading to more precise segmentation contours.
- We conduct extensive experiments demonstrating the effectiveness and efficiency of our approach. Our fully automatic model not only significantly outperforms the standard auto-modes of SAM/SAM2 but also surpasses their GT-Bbox prompted modes. This is achieved with only 22.9M trainable parameters, showcasing a superior balance of performance and efficiency and validating the power of our proposed paradigm.

## 2. Related Work

### 2.1. Visual Backbones for Segmentation

The performance of segmentation models is heavily reliant on the quality of features extracted by their visual backbone. Modern architectures often face a trade-off between capturing fine-grained local details and robust global context.

Hierarchical Vision Transformers, such as Hiera [9], represent a significant advancement in this area. Their strength is twofold. Architecturally, by employing window-based local attention in their early stages, they excel at preserving high-frequency spatial information like edges and textures. Furthermore, their pre-training under the masked autoencoder (MAE) framework [10] is crucial. This self-supervised strategy forces the model to reconstruct randomly masked image patches, compelling it to learn rich and robust local representations without relying on extensive labeled data. This combination of an efficient architecture and a powerful pre-training scheme makes them ideal for tasks requiring precise boundary delineation.

On the other hand, CNN-based architectures, particularly those with a U-shaped structure like U-Net [11], have long been the standard for semantic segmentation. Their design of progressive downsampling to capture semantic information followed by upsampling to recover spatial resolution makes them powerful at abstracting the global context of an image. U^2^-Net [12] further enhances this paradigm with its nested ReSidual U-blocks (RSUs), enabling even deeper feature abstraction across multiple scales. Our work leverages the complementary nature of these two architectural paradigms by designing a novel fusion mechanism to combine their respective strengths.

### 2.2. Advances in Improving SAM

Research on improving SAM has predominantly advanced along four key directions: enhancing its automation, broadening its domain adaptability, refining its output precision, and fostering synergy with other foundation models.

Automated Prompt Generation. The core interactive mechanism of SAM relies on manual prompts, which limits its efficiency in fully automated applications. Consequently, a significant line of research focuses on developing techniques for automatic prompt generation. A mainstream approach involves training an auxiliary network to predict prompt cues like points or boxes [13,14], or refining an initial set of prompts into a more representative sparse point collection [15]. Another strategy leverages the semantic understanding of multimodal large models, such as CLIP [16], to generate guiding signals like pseudo-points, pseudo-masks, textual, or auditory prompts [17,18], providing SAM with initial localization information. However, these methods typically generate sparse prompts (points or boxes) or depend on implicit guidance from external models, limiting their ability to provide a global, structurally rich prior for the segmentation target.

SAM Adaptation and Enhancement. To adapt SAM for specialized domains like medical imaging, remote sensing, and video, research has proceeded along two main avenues: Parameter-Efficient Fine-Tuning (PEFT) and architectural modification. PEFT methods, such as Adapters and Low-Rank Adaptation (LoRA), aim to adjust parameters of SAM with minimal computational overhead [19,20]. Some studies have also designed novel fine-tuning mechanisms, like prompt-bridging, to balance the optimization between the encoder and decoder [21]. Architectural enhancements, on the other hand, focus on tailoring the model to specific data types. This includes introducing temporal modeling modules for video [22,23,24] and integrating state-space models like Mamba [25] to capture domain-specific spatiotemporal dependencies. These works concentrate on adjusting the internal parameters of model or structure to accommodate new tasks or data modalities.

Output Quality Refinement. Addressing the issue of coarse boundaries in output masks of SAM, research has primarily adopted post-processing refinement strategies. This involves introducing an additional network to polish the initial masks generated by SAM [26]. Alternatively, some approaches focus on decoder optimization by fusing multi-scale features or improving feature aggregation methods to enhance the detail quality of the native output [19,20,27]. Such methods are characteristically post hoc, concentrating on correcting or enhancing the segmentation result after it has been generated.

Collaboration with Other Foundation Models. Beyond single-model adaptation, a body of work explores the deep integration of SAM with other large models, notably CLIP, at a systemic level. This includes constructing explicit pipelines, such as using CLIP for localization followed by SAM for segmentation [17], deconstructing SAM and coupling it with a CLIP encoder to create an efficient single-stage open-vocabulary segmentation architecture [15], or utilizing SAM for contextual segmentation while introducing uncertainty modeling to improve robustness [28]. These efforts demonstrate potential of SAM as a versatile component within more complex, multimodal systems.

### 2.3. Positioning of Our Work

Our work carves a distinct and novel path within this research landscape. While existing research has largely focused on two avenues—either enhancing the promptable mode through automated prompt generation or adapting SAM’s internal architecture for specific domains—our work addresses a more fundamental issue: the inherent limitation of the automatic mode for targeted segmentation tasks.

Unlike automated prompt generation methods that aim to replace manual clicks with predicted sparse cues like points or boxes, our Pre-Mask Guided paradigm introduces a new, fully automatic pipeline. It provides a dense, explicit mask rich in spatial and structural priors, shifting the operational model from a “prompt-and-segment” process to an end-to-end “locate-and-refine” workflow. This approach fundamentally solves the localization blindness of the grid-based segment everything approach. In contrast to methods that fine-tune SAM’s parameters or add post-processing steps to refine coarse masks, our framework operates at the input level of the decoder. By furnishing a high-quality shape prior before the main segmentation process begins, we preemptively guide the model towards a precise solution. This makes our approach a foundational, architectural innovation rather than a domain-specific adaptation or a corrective afterthought. In essence, PMG-SAM proposes a new blueprint for building general-purpose automatic segmentation models that are both efficient and target-aware, making it a versatile and powerful component for complex vision systems. In essence, while other methods focus on what to prompt SAM with, our work redefines how SAM’s automatic mode should fundamentally operate. It is a shift from a ‘prompt-and-segment’ philosophy to an integrated ‘locate-and-refine’ workflow, making it an architectural innovation rather than a prompting technique.

## 3. Methods

The native prompt-free mode of SAM is hampered by two fundamental limitations: a localization sensitivity defect and a redundant computation bottleneck. To address these challenges head-on, we propose PMG-SAM, a framework that replaces SAM’s exhaustive grid-based search with a targeted, cognition-inspired “locate-and-refine” paradigm. This section first revisits the architecture and limitations of SAM in Section 3.1, before detailing the design and components of our proposed solution in Section 3.2.

### 3.1. Preliminaries: SAM

#### 3.1.1. Architectural Framework

SAM comprises three core components: an image encoder, a prompt encoder, and a mask decoder.

The image encoder, accounting for the majority of model parameters, employs a Vision Transformer (ViT) [29] pre-trained via Masked Autoencoding (MAE) [10] to process high-resolution inputs, with each image undergoing single-pass feature extraction.

The prompt encoder is a core component that establishes SAM as a multimodal architecture, processing two distinct prompt categories: dense and sparse. Dense prompts, which consist of mask inputs, are processed by a convolutional encoder and their embeddings are added element-wise to the image embeddings. To handle cases where no mask is provided, the model incorporates a learnable “no-mask” embedding. This special token serves as a placeholder to signify the absence of a mask, ensuring the architectural consistency of the model. In contrast, sparse prompts encompass three modalities—points and boxes encoded as positional embeddings combined with modality-specific learnable embeddings, alongside textual prompts processed with CLIP token embedding.

The mask decoder integrates image features and prompt embeddings through bidirectional cross-attention and self-attention mechanisms, subsequently employing a multilayer perceptron (MLP) to project output tokens to a dynamic linear classifier that computes foreground probability masks at each spatial location.

#### 3.1.2. Prompt-Free Segmentation

SAM implements prompt-free segmentation through a two-stage cascade beginning with grid sampling, where a default N×N grid (N=32) is overlaid on the input image and processed at each grid point to generate candidate object masks, with repetition on two cropped high-resolution regions using denser point sampling. This is followed by mask refinement through a three-phase filtering mechanism: (1) elimination of edge-affected masks from cropped regions; (2) application of Non-Maximum Suppression (NMS) for local-global mask merging; and (3) implementation of quality control via three criteria—retention of masks with IoU scores ≥88.0, stability verification through soft mask thresholding at τ={−1,+1} with predictions kept only when $\mathrm{IoU}(M_{-1},M_{+1})\geq 95.0$, and rejection of masks covering ≥95% of the image area as non-informative.

This paradigm reveals two critical limitations. First, it suffers from a localization sensitivity defect, where grid-based dense prompts are vulnerable to semantic fragmentation, leading to erroneous multi-fragment segmentation of single objects. This observation is corroborated by the work of Sun et al. [5], which demonstrates that native geometric prompts of SAM struggle in complex scenes precisely due to a lack of semantic guidance. Second, the paradigm introduces a redundant computation bottleneck. The per-point cross-attention in the ViT decoder, with its $O(N^{2})$ complexity, leads to a computational explosion when high-resolution grids are employed, violating linear complexity expectations. The drive to mitigate such computational inefficiencies is a key motivation in related research, such as the model merging approach proposed by Wang et al. [30] to create a more efficient, unified model.

### 3.2. Architecture

**Figure 2:**
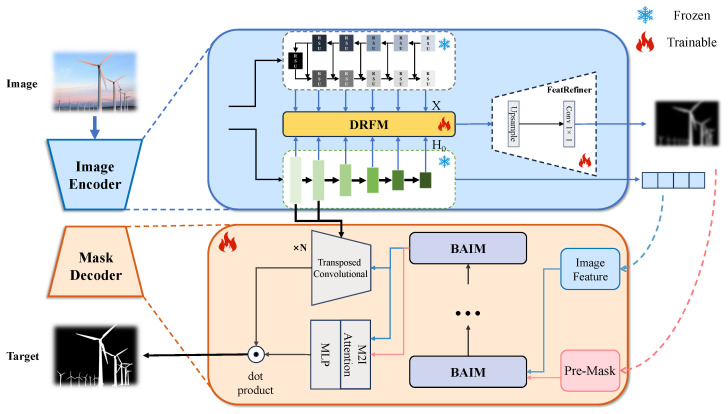
Overview of the PMG-SAM pipeline.

To overcome the inherent limitations of automatic mode of SAM, namely its localization blindness and computational inefficiency, we introduce a Pre-Mask Guided SAM (PMG-SAM). Our approach is inspired by the “coarse-to-fine” cognitive mechanism characteristic of human vision. The overall architecture of PMG-SAM, illustrated in Figure 2, is streamlined and comprises two primary components: a novel image encoder and an enhanced mask decoder. To operationalize our proposed ‘locate-and-refine’ paradigm, the architecture of PMG-SAM is conceptually divided into two functional stages: a Pre-Mask Generator responsible for the ‘locate’ step comprising our dual-branch encoder and fusion modules, and a Guided Mask Decoder that executes the ‘refine’ step which leverages the generated Pre-Mask for precise segmentation.

The core innovation of our framework lies in the Pre-Mask guided paradigm. Specifically, we replace original image encoder of SAM, which relies on a grid of point prompts for automatic mask generation, with our specialized encoder. This new encoder is designed to automatically generate a high-quality, dense Pre-Mask from the input image. The Pre-Mask serves as a global, structural prompt, providing a strong prior about the locations and shapes of potential objects. This Pre-Mask is then fed into our enhanced mask decoder to guide the segmentation process. Crucially, to further boost precision, the decoder is also strategically augmented with high-resolution features extracted by our new encoder. This dual-input design—leveraging both the high-level semantic guidance of the Pre-Mask and the edge detail from the features—enables the production of highly accurate and detailed segmentation masks. By replacing a exhaustive strategy with a learnable, content-aware guidance mechanism, PMG-SAM fundamentally shifts the operational process from exhaustive search to targeted refinement.

To effectively capture the full spectrum of visual information required for high-quality segmentation, our feature extractor is designed with a dual-pathway architecture. This design is motivated by the observation that different architectural paradigms excel at capturing distinct types of features: hierarchical transformers are superior for fine-grained local details, while U-Net-like structures are powerful for abstracting global semantic context. For the local feature pathway, we adopt Hiera as our backbone, replacing the original ViT from SAM. The choice of Hiera is deliberate, owing to its powerful combination of architectural design and pre-training strategy. Its hierarchical structure with local attention is inherently suited for capturing fine-grained details, a capability significantly amplified by its MAE-based pre-training. This training regime compels the model to develop a profound understanding of local patterns and textures, which aligns with findings from [31] on the importance of local features for precise segmentation. Concurrently, for the global feature pathway, we employ a U^2^-Net-like architecture. Its deeply supervised, nested U-structure is highly adept at progressive semantic abstraction, yielding robust global feature representations that capture the overall structure and context of target objects.

The core novelty of our approach lies not in the individual backbones, but in their synergistic fusion. To combine the complementary strengths of local acuity of Hiera and the U^2^-Net pathway’s global understanding, we introduce our Dense Residual Fusion Module (DRFM). This module systematically integrates feature maps from both pathways. The process involves three key stages: extraction of $N_{1}$ corresponding feature maps from both architectures, where $N_{1}$ is set to six by default.; pairwise feature fusion with DRFM (detailed in Section 3.3); processing of fused features through FeatRefiner (detailed in Section 3.4).

### 3.3. Dense Residual Fusion Module

Dense Residual Fusion Module (DRFM) is designed to effectively merge the strengths of the Hiera and U^2^-Net feature streams. We adopt a layer-wise fusion strategy, where features from corresponding layers of the two models are fused pairwise. This approach is rooted in the belief that integrating local, detail-oriented features (from Hiera) with multi-scale semantic features (from U^2^-Net) at the same hierarchical level ensures meaningful feature interaction. In contrast to a global fusion approach, this targeted strategy prevents the mixing of features from disparate semantic levels, which could lead to information misalignment and redundant interference. Furthermore, by confining interactions to corresponding layers, we significantly reduce computational overhead.

**Figure 3:**
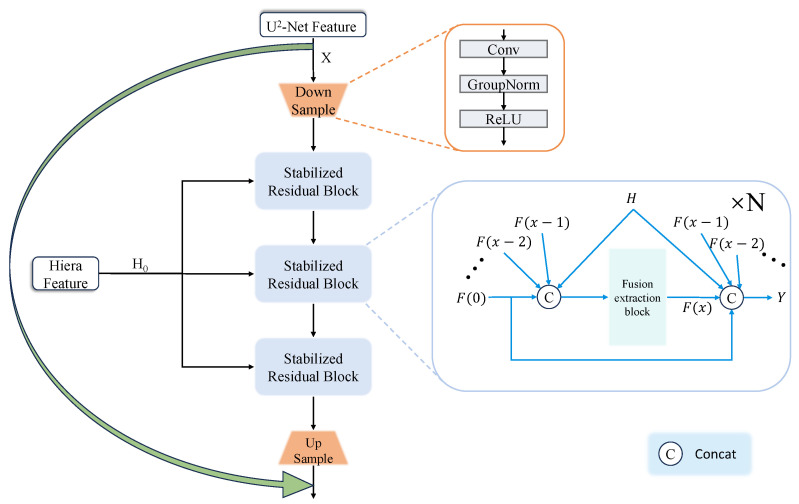
Schematic of the DRFM structure. Details of the fusion extraction block are provided in Algorithm 2.

As depicted in Figure 3, the operational flow of DRFM designates a feature map from a U^2^-Net layer (U^2^-Net Feature) as the primary backbone for fusion. This feature is progressively refined by passing through a cascade of $N_{2}$ Stabilized Residual Blocks (SRBs), where $N_{2}$ is set to three by default. Within each SRB, the corresponding feature map from the Hiera model (H-Feature) is integrated. Finally, the output from the SRB cascade is scaled and added back to the original U^2^-Net Feature via a long-range skip connection. As previously discussed, this process enriches the robust global context from U^2^-Net with high-fidelity local details from Hiera. Consequently, the fused features facilitate the generation of a pre-mask that possesses both precise localization and complete edge information, thereby providing superior guidance for the mask decoder to extract a highly refined mask of the object of interest. The overall fusion process of DRFM is summarized in Algorithm 1.
**Algorithm 1:** The Overall Pipeline of DRFM.
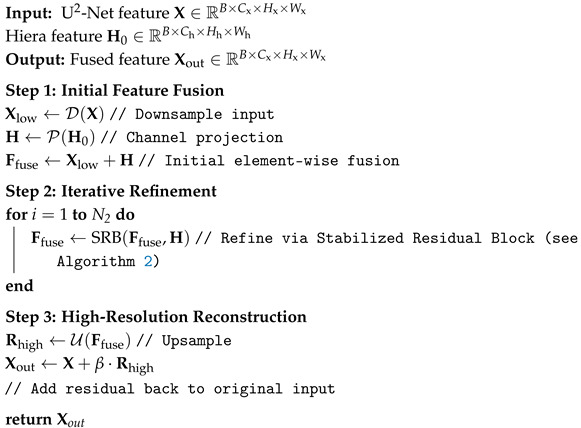


The internal architecture of the Stabilized Residual Block (SRB) is also illustrated in Figure 3. An SRB takes an input feature $F_{0}$ and the corresponding H-Feature *H*. It performs *M* iterations of residual fusion, with *M* defaulting to four in our implementation. A key characteristic of this process is its dense connectivity: the new feature at each iteration is derived from a residual connection involving all preceding features in the block as well as the H-Feature *H*. This mechanism is termed a “Dense Residual”. The total accumulated residual from these iterations is then scaled and added to the original input $F_{0}$, followed by a Group Normalization layer to produce the final output *Y*. This scaling of the residual value, applied in both the overarching DRFM structure and within each SRB, serves as a crucial stabilization technique to prevent gradient explosion during training. The internal fusion process of the SRB is detailed in Algorithm 2.
**Algorithm 2:** The details of SRB.
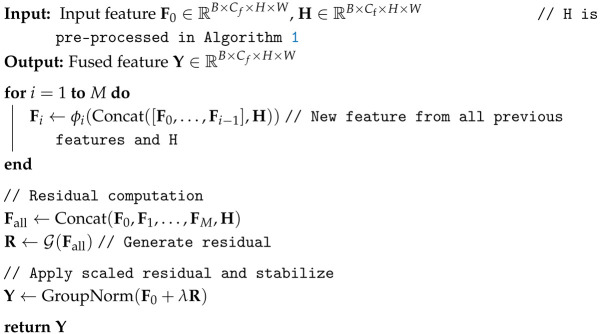


### 3.4. FeatRefiner

The FeatRefiner module is designed with the explicit purpose of processing the multi-scale, hierarchical feature maps generated by the preceding DRFM fusion stages. Its primary function is to distill these diverse representations into a single, cohesive Pre-Mask. The refinement process commences by upsampling all fused feature maps from the different layers to a uniform spatial resolution. This ensures spatial alignment before integration. Subsequently, the resized feature maps are concatenated along the channel axis, creating a high-dimensional composite tensor that aggregates rich information from all semantic levels. To conclude the process, a lightweight 1×1 convolutional layer is applied to this concatenated tensor. This operation serves the dual purpose of compressing the channel-wise information and effectively extracting the most salient features from the aggregated representation. The final output of this module is the Pre-Mask, denoted as $M_{\mathrm{pre}}\in R^{B\times 1\times H\times W}$, which provides a comprehensive initial localization of the object of interest to guide the subsequent mask decoder.

### 3.5. Mask Decoder

The outputs from the image encoder are subsequently processed by our enhanced mask decoder. This decoder is architecturally defined by a cascade of $N_{3}$ Bidirectional Attention Interaction Modules (BAIMs), which iteratively refine the representations. As illustrated in the overall framework (Figure 2), upon passing through the BAIM stack, the refined image embeddings undergo upsampling with transposed convolutions. Critically, to enhance the generation of high-resolution details, we inject fine-grained feature maps from the initial two stages of the Hiera encoder directly into these transposed convolution layers. Unlike standard ViT features which may lose high-frequency details, the early stages of Hiera, benefiting from its local attention mechanism and MAE pre-training, preserve rich spatial texture and edge information. These high-resolution features are rich in detailed edge information, proving vital for producing crisp and accurate segmentation boundaries. Finally, an MLP maps the output tokens to a dynamic linear classifier, and the foreground probability for each pixel location is computed with a dot product to yield the final mask.

**Figure 4:**
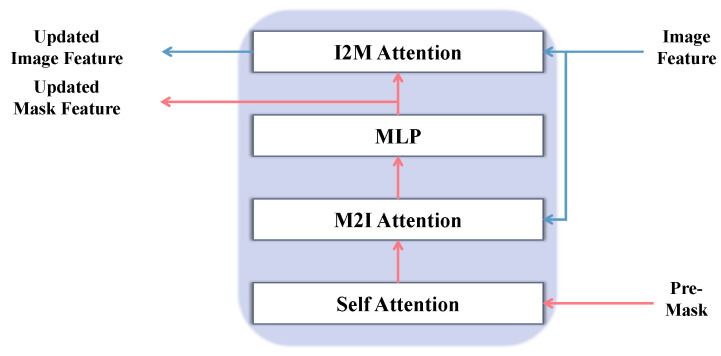
Bidirectional Attention Interaction Module (BAIM).

The internal architecture of the BAIM, detailed in Figure 4, is meticulously designed to facilitate a comprehensive, bidirectional information flow. The input Pre-Mask is first partitioned into a grid of non-overlapping patches of size P×P (where P=16 by default) and linearly embedded to form a sequence of $P^{2}$ mask tokens. The processing pipeline within a single BAIM block commences with a self-attention layer applied to these mask tokens, enabling them to model their internal spatial dependencies. Following this intra-mask modeling, the refined tokens serve as queries in a Mask-to-Image (M2I) cross-attention mechanism, attending to the image features to gather contextually relevant visual information. Each token is then independently updated by a pointwise MLP block. The bidirectional interaction culminates in an Image-to-Mask (I2M) cross-attention layer. In this crucial step, the roles are inverted: the image features now act as queries to attend to the updated mask tokens, allowing the image representation itself to be refined based on the focused guidance from the mask. This complete cycle of self-attention, dual cross-attention, and MLP-based updates constitutes one interaction block, and its outputs are passed to the subsequent BAIM for further iterative refinement, ensuring the guidance from the Pre-Mask is fully leveraged.

## 4. Experiment

In this section, we present a comprehensive set of experiments to empirically validate our central thesis: that the Pre-Mask Guided “locate-and-refine” paradigm represents a superior architectural solution for automatic segmentation compared to SAM’s native “segment everything” approach. We conduct our evaluation on two distinct tasks that represent the opposing extremes of visual perceptibility: Dichotomous Image Segmentation (DIS) which characterized by high saliency but complex structure and Camouflaged Object Segmentation (COS) which characterized by low saliency and texture ambiguity. Validating on these extremes ensures the model’s robustness across the full spectrum of segmentation challenges. We begin by introducing the datasets and evaluation metrics employed for each task. Subsequently, we describe the implementation details, including the experimental environment, model configuration, and the specific training and inference procedures. We then report and analyze the quantitative results on the DIS5K [2], COD10K [32], and NC4K [33] benchmark datasets, comparing our method against other approaches. Finally, we conduct extensive ablation studies to investigate the impact of individual components on the overall performance.

SAM-B, SAM-L, and SAM-H represent ViT-B, ViT-L, and ViT-H model types of SAM, respectively. SAM2-T, SAM2-B+, and SAM2-L represent Hiera-Tiny, Hiera-Base+, and Hiera-Large model types of SAM2, respectively.

### 4.1. Datasets and Evaluation Metrics

#### 4.1.1. DIS Task: Dataset and Metrics

Our experiments use the DIS-5K dataset, the first large-scale benchmark specifically designed for high-resolution (2K, 4K, and beyond) binary image segmentation. The dataset consists of 5470 meticulously annotated images, organized into 22 groups across 225 categories. These images include diverse objects camouflaged in complex backgrounds, salient entities, and structurally dense targets. Each image was manually annotated at the pixel level, with an average labeling time of 30 min per image, extending up to 10 h for particularly complex instances. According to the official partition, the dataset is divided into 3000 training images(DIS-TR), 470 validation images(DIS-VD), and 2000 test images. The test set is further divided into four subsets (DIS-TE1 to DIS-TE4), each containing 500 images, representing ascending difficulty levels based on the product of structural complexity (IPQ) and boundary complexity ($P_{num}$).

Evaluation uses six established metrics: maximal F-measure ($F_{\beta }^{\max}↑$) [34], weighted F-measure ($F_{\beta }^{\omega }↑$) [35], Mean Absolute Error (M↓) [36], Structural measure ($S_{\alpha }↑$) [37], mean Enhanced alignment measure ($E_{\phi }^{m}↑$) [38], and Human Correction Efforts ($HCE_{\gamma }↓$) [2]. Arrows indicate the preferred direction (↑: higher is better, ↓: lower is better).

The maximal F-measure evaluates the optimal trade-off between precision and recall. It calculates the F-measure scores across varying thresholds (τ) and selects the maximum value. Following the standard setting in saliency detection, we set $\beta^{2}=0.3$ to emphasize precision. It can be expressed as

(1) $$F_{\beta }^{\max}=\max_{\tau }\left(\frac{\left(1+\beta^{2}\right)\cdot \mathrm{Precision}\left(\tau \right)\cdot \mathrm{Recall}\left(\tau \right)}{\beta^{2}\cdot \mathrm{Precision}\left(\tau \right)+\mathrm{Recall}\left(\tau \right)}\right).$$

The weighted F-measure ($F_{\beta }^{w}$) utilizes a weighted precision ($Precision^{w}$) and weighted recall ($Recall^{w}$) to address the flaw that standard measures treat all pixels equally. It considers the spatial dependence and pixel importance. In our evaluation, we set $\beta^{2}=1$. It can be expressed as

(2) $$F_{\beta }^{w}=\frac{\left(1+\beta^{2}\right)\cdot {\mathrm{Precision}}^{w}\cdot {\mathrm{Recall}}^{w}}{\beta^{2}\cdot {\mathrm{Precision}}^{w}+{\mathrm{Recall}}^{w}}.$$

The Mean Absolute Error quantifies the global accuracy of predictions by computing the average absolute difference between the predicted map (Pred) and the ground-truth map (GT) across all pixels. A lower MAE indicates higher consistency between predictions and ground truths. It can be expressed as

(3) $$\mathrm{MAE}=\frac{1}{N}\sum_{i,j}\left|\mathrm{Pred}(i,j)-\mathrm{GT}(i,j)\right|.$$

The Structural measure (S-measure) simultaneously evaluates the region-aware structural similarity ($S_{r}$) and object-aware structural similarity ($S_{o}$) between the prediction and ground truth. With the balance parameter α set to 0.5. It can be expressed as

(4) $$S_{\alpha }=\alpha \cdot S_{o}+\left(1-\alpha \right)\cdot S_{r}.$$

The mean Enhanced alignment measure calculates the average *E*-value (which integrates pixel-level and region-level errors) across multiple thresholds ($\tau_{t}$), reflecting error distribution characteristics at both local and global scales. It can be expressed as

(5) $$E_{\phi }^{m}=\frac{1}{T}\sum_{t=1}^{T}E\left(\tau_{t}\right).$$

The Human Correction Efforts is a metric designed to quantify the barriers between model predictions and real-world applications. Unlike standard metrics that measure the geometric gap (e.g., IoU), HCE approximates the human efforts required to correct the faulty regions (False Positives and False Negatives) in a segmentation mask. Specifically, it estimates the number of mouse clicking operations needed for correction, including dominant point selection for boundary refinement and region selection for area fixing. A lower $HCE_{\gamma }$ value indicates a reduced manual revision workload, signifying that the model’s output satisfies high-accuracy requirements with fewer human interventions. It can be expressed as

(6) $$HCE_{\gamma }=\frac{1}{K}\sum_{k=1}^{K}\left({\mathrm{FP}}_{\mathrm{points}}^{\left(k\right)}+{\mathrm{FP}}_{\mathrm{indep}}^{\left(k\right)}+{\mathrm{FN}}_{\mathrm{points}}^{\left(k\right)}+{\mathrm{FN}}_{\mathrm{indep}}^{\left(k\right)}\right).$$

#### 4.1.2. COS Task: Datasets and Metrics

For the COS task, our experiments are based on the COD10K and NC4K datasets. COD10K is the first large-scale benchmark for camouflaged object detection, across 78 sub-classes and 10 super-classes, capturing diverse camouflage scenarios in natural environments. We follow the official split of 3040 images for training and 2026 for testing. The NC4K dataset, containing 4121 images of camouflaged objects in nature, serves as a supplementary benchmark to validate the generalization capability of the models.

To evaluate segmentation performance, we adopt the standard COCO-style metrics: Average Precision (AP), $AP_{50}$, and $AP_{75}$.

Average Precision (AP) is the primary metric, calculated as the mean of APs over multiple IoU (Intersection over Union) thresholds (from 0.5 to 0.95 with a step of 0.05). It provides a comprehensive measure of instance segmentation quality. $AP_{50}$ and $AP_{75}$ are variants of AP calculated at single, fixed IoU thresholds of 0.5 and 0.75, respectively. $AP_{50}$ evaluates basic detection and localization accuracy, while $AP_{75}$ imposes a stricter criterion for more precise mask predictions.

### 4.2. Experiment Settings

Our proposed PMG-SAM is an enhanced architecture based on the SAM. To enhance the multi-scale feature representation, we employ a Feature Pyramid Network (FPN) [39] to process the features extracted by the Hiera encoder before they are fed into the mask decoder. Our performance is benchmarked against numerous leading models, including SAM2 [40], a recent advancement for both image and video segmentation. All experiments were conducted on a system running Ubuntu 20.04.6, equipped with a single NVIDIA Tesla A100-PCIE-40GB GPU (NVIDIA, Santa Clara, CA, USA), and built upon a stack of PyTorch 2.5.1, CUDA 11.8, and Segment-Anything 1.0.

### 4.3. Training and Inference Procedure

To leverage powerful prior knowledge and accelerate training, the image encoder components—Hiera and U^2^-Net—are initialized with pre-trained weights and remain frozen during training. To ensure computational efficiency and prevent overfitting on limited downstream data, the image encoder components—Hiera and U^2^-Net—remain fully frozen throughout the training process. The Hiera-base+ model was pre-trained on the ImageNet-1K dataset [41] using MAE self-supervised learning framework. The U^2^-Net component uses weights pre-trained on the DUTS dataset [42]. The total loss function is a weighted sum of three standard segmentation losses: Binary Cross-Entropy (BCE), Dice, and IoU loss. The total loss $L_{\mathrm{all}}$ is computed as:

(7) $$L_{\mathrm{all}}=w_{1}L_{\mathrm{BCE}}+w_{2}L_{\mathrm{Dice}}+w_{3}L_{\mathrm{IoU}}.$$

where the weights $w_{1},w_{2},w_{3}$ are set to 1, 1, and 10. Following the original SAM, the number of BAIM, $N_{3}$, is set to 2. The batch size is set to 4. For data preprocessing, input images undergo a series of augmentations, including random cropping, random horizontal flipping, random rotation between $-30^{\circ }$ and $30^{\circ }$, and random color jittering. Subsequently, all images are resized and padded to a fixed resolution of 1024×1024 to comply with the input requirements of the Hiera encoder.

For the DIS task, the model is trained for 300 epochs. We use the AdamW optimizer [43] with an initial learning rate of $1\times 10^{-3}$, a weight decay of 0.1, and momentum of 0.9. A learning rate warm-up period is applied for the first 10 epochs, followed by a decay schedule where the learning rate is reduced by 40% every 40 epochs. An early stopping mechanism is in place, terminating the training if the validation loss does not improve for 40 consecutive epochs.

For the COS task, we fine-tune the model using the best-performing weights obtained from the DIS task. We conduct two separate fine-tuning processes: one on the COD10K training set and another on the NC4K training set. The NC4K dataset is first partitioned into training (60%) and testing (40%) sets. In both fine-tuning pipelines, the respective training set is further split into an 85% training subset and a 15% validation subset. The initial learning rate is set to a lower value of $1\times 10^{-5}$. We employ a learning rate scheduler that reduces the learning rate by half if the validation loss plateaus for 5 consecutive epochs, with a minimum learning rate of $1\times 10^{-7}$. The optimizer remains AdamW with a weight decay of 0.1. The model is trained for a total of 90 epochs, with a 10-epoch warm-up and an early stopping patience of 20 epochs.

During inference, the 1024×1024 input image is passed through PMG-SAM to generate a binary segmentation map. For the DIS task, the inference is fully end-to-end and requires no post-processing. For the COS task, a specific post-processing pipeline is employed to separate potentially overlapping or adjacent objects. First, during training, the multiple instance masks from the COD10K ground truth are merged into a single binary mask, guiding the model to learn the general concept of a “camouflaged object”. At inference time, we apply Connected Component Analysis (CCA) [44] to the model’s binary output to separate the binary mask into individual object proposals. The Hungarian algorithm [45] is then used to perform one-to-one matching between the predicted instances and the ground-truth masks. Finally, the matched predictions are saved in the standard COCO JSON format to enable evaluation with the AP metrics.

### 4.4. Results on DIS Task

We begin our analysis with the DIS task, which serves as the primary benchmark to evaluate the core segmentation quality of our model against baselines and specialized methods. The fine-grained and complex nature of objects in the DIS5K dataset provides an ideal testbed to assess the efficacy of our approach.

#### 4.4.1. Efficiency and Complexity Analysis

Table 1 presents a comprehensive comparison of model complexity and inference efficiency. A key advantage of our approach is its superior balance between parameter efficiency and practical speed.

First, regarding parameter efficiency, PMG-SAM requires only 22.9 M trainable parameters, making it significantly more lightweight to train than even the smallest SAM2-T model (38.9 M).

Second, we address the concern regarding computational cost. Although our total FLOPs (1116.2 G) are relatively high due to the utilization of powerful frozen backbones (Hiera-B+ and U^2^-Net), our method achieves the highest inference speed of 4.85 FPS. This result reveals a critical insight: the standard “automatic mode” of SAM and SAM2 relies on a dense grid-prompting strategy, which suffers from severe computational redundancy and slows down inference. In contrast, our “locate-and-refine” paradigm generates a global prior in a single pass, avoiding exhaustive grid search. Thus, despite higher theoretical FLOPs per pass, our actual wall-clock inference time is significantly lower.

Finally, regarding memory consumption, PMG-SAM operates with a peak memory of approximately 3420 MB. This is comparable to the base-sized models and significantly lower than the large variants (e.g., SAM-H requires 5731 MB), ensuring deployability on standard GPUs.

#### 4.4.2. Quantitative Results

We extensively evaluated PMG-SAM against baseline models (SAM, SAM2) and specialized methods including HRNet [46], STDC [47], IS-Net [2], and SINetV2 [48] across DIS-VD and DIS-TE1–4 datasets. Quantitative results on DIS5K validation and test sets (Table 2) reveal consistent improvements across all metrics.

Table 2 presents the quantitative comparison of our method against both baseline and task-specific models on the DIS5K dataset. PMG-SAM demonstrates a comprehensive improvement across multiple evaluation metrics on both the validation and test sets. Notably, the HCE value of PMG-SAM is substantially lower than that of the baseline models, which not only signifies a numerical superiority but also reflects the targeted design of our architecture to address key segmentation challenges. Our model not only achieves significant gains over the baselines but also rivals and even surpasses the performance of methods specifically designed for the DIS task.

#### 4.4.3. Analysis Against Baseline Models

Our analysis against baseline models is structured to answer two key questions: (1) How effective is our framework at overcoming the limitations of SAM’s original “segment everything” automatic mode? (2) Is our approach merely an automated prompter, or does it represent a fundamentally more powerful segmentation system?

As shown in Table 2, when compared to the standard Auto mode of all SAM and SAM2 variants, PMG-SAM demonstrates a transformative leap in performance. For instance, on the challenging DIS-TE2 set, our model achieves a maximal F-measure of 0.815, a stark contrast to the 0.283 of SAM-H and 0.442 of SAM2-L. This massive improvement across all metrics confirms that our Pre-Mask Guided paradigm effectively solves the localization blindness and fragmentation issues inherent in the grid-based approach, enabling precise and coherent segmentation of target objects without human intervention.

Crucially, to explicitly validate whether the localization failure of SAM stems from a lack of domain knowledge, we compared PMG-SAM against a fine-tuned version of SAM-H (the largest variant). As shown in Table 2, although fine-tuning improves the performance of SAM-H in automatic mode (raising $F_{\beta }^{max}$ from 0.283 to 0.378 on DIS-VD), it still lags significantly behind our PMG-SAM ($F_{\beta }^{max}$ 0.791). This substantial gap confirms that parameter optimization alone cannot resolve the inherent “localization blindness” of the grid-search strategy. In contrast, our Pre-Mask paradigm provides the necessary structural prior, achieving superior performance with significantly fewer trainable parameters.

To further investigate the effectiveness of our paradigm, we compare our fully automatic method against baseline models guided by ground-truth bounding boxes (GT-Bbox). This represents an ideal scenario for prompt-based methods. As shown in the tables, our PMG-SAM consistently outperforms these perfectly prompted baselines on challenging test sets (e.g., DIS-TE2, TE3, TE4). This finding is particularly insightful. It suggests that the dense, structural information provided by our internally generated Pre-Mask offers a richer and more effective guidance signal to the decoder than a sparse bounding box. This validates that our ‘locate-and-refine’ approach creates a more capable segmentation pipeline, going beyond simple prompt automation.

In summary, these comparisons, combined with the model size analysis in Table 1, robustly demonstrate that PMG-SAM’s performance gains stem from a superior and more efficient architectural paradigm, not merely model scaling or automated prompting. It charts a new path for creating truly automatic and highly accurate general-purpose segmentation models.

#### 4.4.4. Analysis Against Specialized Methods

When compared with other classic DIS methods, our model also exhibits strong competitiveness. Commendably, PMG-SAM’s performance matches and in some metrics exceeds that of IS-Net, the established baseline for the DIS task, showcasing its powerful capabilities. Nevertheless, the Maximum F-measure on certain test sets still has room for improvement compared to IS-Net, which provides a clear direction for our future work. Therefore, while IS-Net stands as a strong and classic baseline specifically designed for the DIS task, our PMG-SAM demonstrates highly competitive performance. This is noteworthy because PMG-SAM is not a task-specific model but rather a general-purpose paradigm for enhancing foundation models. Its strong performance on this challenging benchmark showcases the effectiveness and potential for broader applicability of our approach.

### 4.5. Results on COS Task

To rigorously evaluate the zero-shot transfer capability, transfer learning efficacy, and domain generalization capacity of PMG-SAM, we conducted comprehensive experiments against the baseline SAM alongside state-of-the-art models including SAM2, Mask R-CNN [49], PointSup [50], Tokencut [51], Cutler [52], and TPNet [53]. Benchmarking was performed on the COD10K test set and NC4K dataset.

As shown in Table 3, we first assess the zero-shot transfer capability, a key feature of the SAM series. By directly testing our best DIS-trained model on COD10K and NC4K, we find that PMG-SAM surpasses all SAM2 variants and the unsupervised task-specific method on the COD10K test set. On NC4K, it even matches or exceeds some weakly supervised methods. Although a performance gap with the original SAM-H remains, these results demonstrate that PMG-SAM learns effective and transferable representations from the DIS task, and its generalization ability is stronger than methods that do not rely on any labels.

Next, we performed transfer learning by fine-tuning the best DIS model on the COD10K training set and the NC4K training set. As seen in the “Transfer Learning” section of Table 3, the AP score on COD10K shows a remarkable surge from 12.9 to 30.0, closely approaching the performance of the fine-tuned SAM-H (33.7).

Finally, we tested the domain generalization of the fine-tuned model on the COD10K testing set and the NC4K testing set. The results for domain generalization—evaluated by testing the model on a dataset it was not trained on (e.g., testing the COD10K-trained model on the NC4K test set)—are particularly striking. The AP score reaches 40.3, and the stricter $AP_{75}$ metric hits 41.6, surpassing all compared SAM and SAM2 variants, and even the fully supervised Mask R-CNN baseline.

Furthermore, we included a comparison with the fine-tuned SAM-H. On the COD10K dataset, the fine-tuned SAM-H achieves the highest AP of 39.2, surpassing our transfer learning result. This is expected given SAM-H’s massive parameter count compared to our model. However, on the NC4K dataset, our PMG-SAM in the ‘Transfer Learning’ setting achieves an AP of 39.7, outperforming the fine-tuned SAM-H. This result highlights a key advantage of our architecture: “locate-and-refine” paradigm learns a robust, class-agnostic notion of objectness that generalizes better to unseen distributions.

Collectively, these three sets of experiments demonstrate that our model, pre-trained on the DIS dataset, acquires a powerful foundational segmentation capability. The fact that its performance can be elevated to a state-of-the-art level on new domains with only minimal fine-tuning underscores its excellent domain generalization ability.

This robust performance across diverse settings provides strong evidence that our “locate-and-refine” paradigm is not a narrow, task-specific trick. Instead, it endows the model with a powerful and generalizable foundational segmentation capability. The fact that this capability can be efficiently transferred to new domains to achieve good results positions PMG-SAM as a new and effective blueprint for building the next generation of versatile, fully automatic segmentation models.

#### Qualitative Analysis

**Figure 5:**
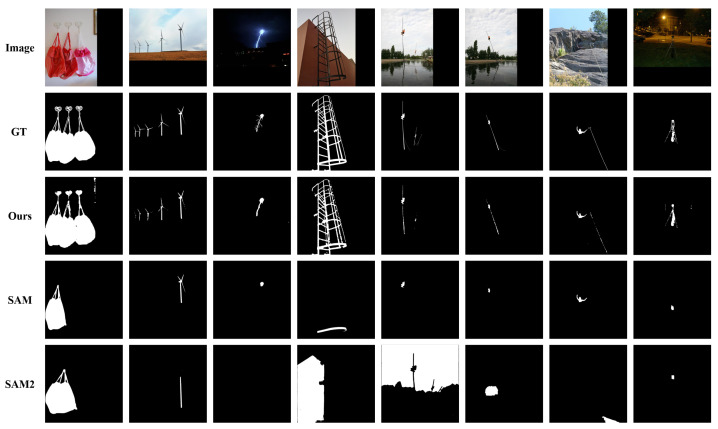
Qualitative comparisons on DIS5K dataset for dichotomous image segmentation.

**Figure 6:**
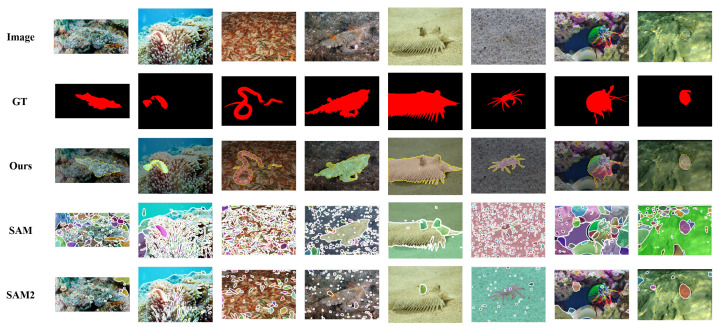
Qualitative comparisons on COD10K and NC4K datasets for Camouflaged Object Segmentation.

To provide an intuitive understanding of our model’s capabilities, we present a series of visual comparisons in Figure 5 for the DIS task and Figure 6 for the COS task. As shown in Figure 5, PMG-SAM exhibits a marked superiority over baseline models across various challenging scenarios in the DIS5K dataset. First, in scenes with complex backgrounds or multiple objects (e.g., columns 1 and 2), our method accurately segments the contours of the bag and wind turbine, whereas the baseline fails to identify the objects completely. Second, our model demonstrates a clear advantage in edge handling. For regions with intricate details, such as the lightning in column 3, PMG-SAM captures the fine edges with high fidelity, while the baseline suffers from significant omissions. Third, our method shows stronger background suppression. In column 5, where the background contains multiple distracting elements, PMG-SAM remains focused on the primary target, unlike the baseline, which is disturbed by background noise. Finally, for small or low-contrast objects, such as the faint support structure in column 8, our model successfully identifies and segments it, a task where the baseline fails. In summary, these qualitative results corroborate our quantitative findings, proving that PMG-SAM is a more robust and precise solution for fully automatic segmentation, particularly in complex scenes.

The qualitative results for the COS task in Figure 6 further underscore the advantages of our method. When dealing with challenging camouflaged targets, PMG-SAM consistently outperforms SAM and SAM2. For instance, it successfully preserves the slender legs of the crab (column 6), which are entirely missed by the baselines. Similarly, it produces a single, coherent mask for the sea snake (column 3), while the baseline outputs are fragmented. These examples highlight superior ability of our model to understand global context and perceive boundaries, enabling it to generate far more accurate and structurally complete masks. This provides compelling visual evidence for the effectiveness and generalization capability of our Pre-Mask-Guided segmentation mechanism.

To further demonstrate the robust localization capability of PMG-SAM across diverse visual domains, we provide additional qualitative results on medical (Kvasir-SEG) and industrial defect (MVTec AD) datasets in Appendix B. These zero-shot inference results confirm that our Pre-Mask mechanism can effectively locate salient targets even in domains completely unseen during training.

### 4.6. Limitation and Future Work

**Figure 7:**
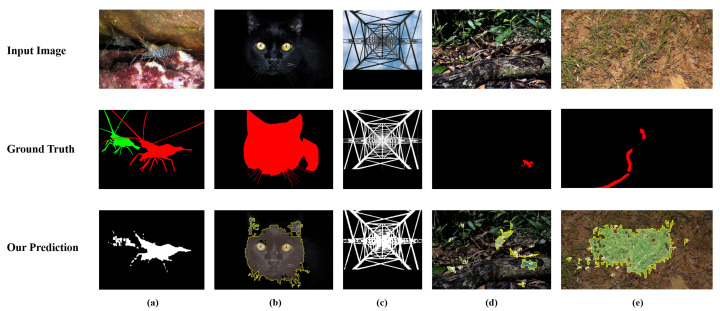
Visualization of typical failure cases categorized by challenge type. From left to right: (**a**) Overlapping Instances, where adjacent objects are merged; (**b**) Low Illumination, where low contrast leads to boundary leakage; (**c**) Complex Topology, where fine structural grids are lost; (**d**) Tiny Objects, where small targets are missed; and (**e**) Complex Texture, where foreground-background similarity causes fragmentation.

To systematically analyze the boundaries of PMG-SAM, we evaluated the model under extreme scenarios. As illustrated in Figure 7, we categorize the primary failure cases into four distinct types, revealing different underlying limitations:

First, Instance Distinctions (Column a): In scenarios with overlapping instances (e.g., the shrimp), the model correctly identifies the salient region but merges adjacent objects into a single connected component. This explicitly exposes a fundamental limitation of our current architecture: since the Pre-Mask paradigm focuses on generating a high-quality binary prior, it relies on post-processing (CCA) rather than a genuine end-to-end mechanism to distinguish instances. Consequently, without instance-specific queries or a separate mask head, the model struggles to separate topologically connected instances.

To strictly quantify this limitation, we filtered the COD10K and NC4K test sets to create specific ‘Overlapping Subsets.’ Statistical analysis reveals that overlapping instances are relatively rare, accounting for only 6.61% (134/2026) of COD10K and 5.68% (234/4121) of NC4K. However, on these specific subsets, the performance of PMG-SAM drops significantly. As shown in the supplementary analysis, the model achieves an AP of only 0.060 on the COD10K overlapping subset and 0.098 on the NC4K overlapping subset. Compared to the overall performance (AP 30–40%), this drastic degradation confirms that the non-end-to-end reliance on CCA is insufficient for complex instance separation, marking a clear boundary of our current architecture.

Second, Environmental Constraints (Column b): Under low illumination conditions (e.g., the black cat), the reduced contrast gradient between the object and the background impedes the Pre-Mask Generator’s ability to capture precise boundaries, leading to noisy and overflowing edges.

Third, Structural and Scale Challenges (Columns c and d): For objects with complex topologies (e.g., the transmission tower) or tiny scales (e.g., the frog), the downsampling operations in the visual encoder inevitably result in the loss of high-frequency spatial details. This causes fine grid structures to disappear and tiny objects to be missed or blurred in the final mask.

Fourth, Texture Ambiguity (Column e): When dealing with strong camouflage where the foreground texture is statistically nearly identical to the background (e.g., the snake), the model suffers from “feature confusion,” resulting in severe fragmentation of the segmentation map.

These findings point to two clear directions for future work: enhancing the high-resolution feature preservation in the encoder to handle complex structures and tiny objects, and replacing the post hoc CCA with an end-to-end instance-aware mechanism to fundamentally resolve the limitation in separating overlapping targets.

### 4.7. Ablation Study

In this section, we conduct a series of ablation studies to dissect the core mechanisms of PMG-SAM and rigorously evaluate the individual contributions of its key components.

#### 4.7.1. Experimental Design

All ablation studies are conducted on the combined DIS-TE1-4 test sets. We use the full suite of six metrics ($S_{\alpha }$, $F_{\beta }^{\max}$, $F_{\beta }^{\omega }$, $E_{\phi }^{m}$, M, ${\mathrm{HCE}}_{\gamma }$) for a comprehensive evaluation. All experiments share the same training environment and hyperparameters to ensure a fair comparison.

#### 4.7.2. Analysis of Key Components of PMG-SAM

Table 4 presents comprehensive ablation study results to verify the effectiveness of each key component in our PMG-SAM. The study is organized into two main groups: (a) evaluating the effectiveness of our proposed Dual-branch Refined Fusion Module (DRFM), and (b) examining the impact of introducing high-resolution features from different sources.

**Figure 8:**
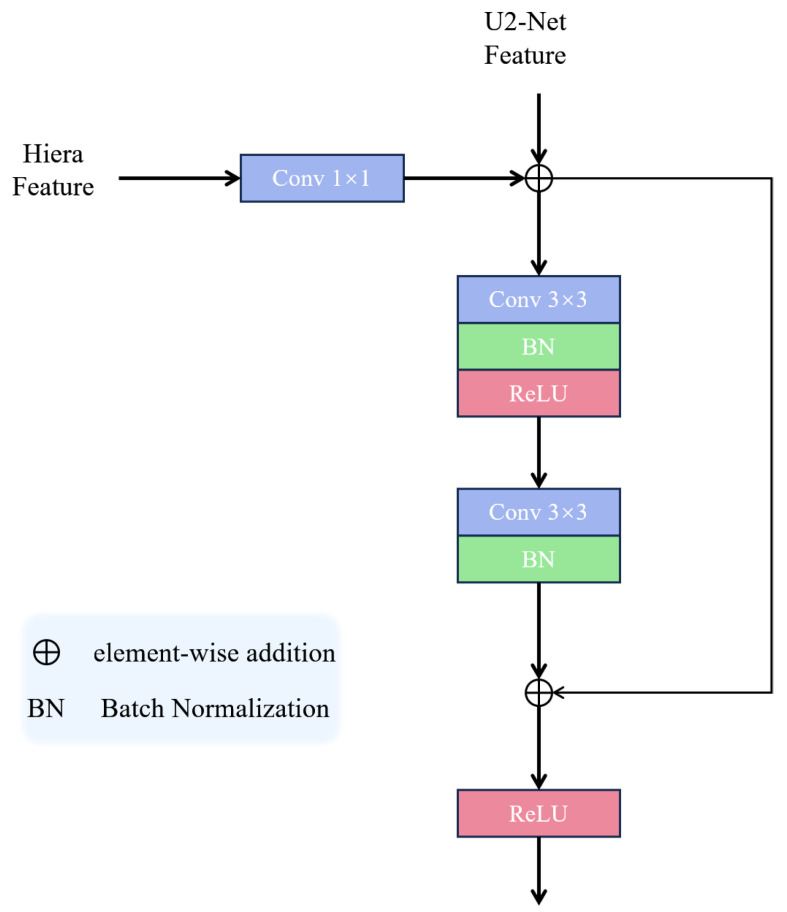
Architecture of the “Residual Fusion” variant used in ablation study (see Table 4).

(a) Feature Fusion Module: In this group, we validate the necessity of our carefully designed DRFM by comparing it with no feature fusion and a simple residual fusion alternative. The results reveal a critical insight: naive feature fusion is detrimental. The Residual Fusion variant, which performs simple element-wise addition followed by a Conv-BN-ReLU block (see Figure 8), achieves significantly worse performance ($F_{\beta }^{\max}$: 0.7329) than both our DRFM-equipped model (0.7974) and even the baseline with no fusion at all (0.7771).

This strongly suggests that due to the vast architectural and feature distribution differences between Hiera and U^2^-Net, direct addition introduces conflicting information and noise, corrupting the original feature representations. In contrast, our DRFM, with its sophisticated structure, successfully aligns and enhances these heterogeneous features, leading to consistent improvements across all metrics.

This leads to another key insight: the quality and relevance of high-resolution features are more important than their mere presence. Our results show that selectively injecting high-fidelity features (from Hiera) is the optimal strategy for boundary refinement.

(b) High-Resolution Feature Strategy: This group identifies the optimal strategy for supplementing the mask decoder with high-resolution features. We examine three approaches: introducing no high-resolution features, only from first two stages of U^2^-Net, or from both Hiera and U^2^-Net backbones. The results offer another profound insight: not all high-resolution features are beneficial.

Introducing only U^2^-Net’s features degrades performance ($F_{\beta }^{\max}$: 0.7409) compared to the baseline without any high-res features (0.7584), indicating that shallow U^2^-Net features may contain excessive background noise or task-irrelevant details. The variant using features from both backbones (0.7769) shows improved performance, suggesting that high-quality of Hiera features can partially offset the negative impact of U^2^-Net’s features.

This result strongly corroborates our architectural analysis in Section 3.2: U^2^-Net features are rich in semantic context but noisy for fine details, whereas Hiera features are structurally precise. Therefore, selectively injecting high-fidelity features (from Hiera) is the optimal strategy for boundary refinement, validating the necessity of this specific dual-backbone design.

Full Model Performance: Our final PMG-SAM model combines DRFM with high-resolution features from Hiera only, achieving the best overall performance across all metrics. This configuration yields the highest scores in $F_{\beta }^{\max}$ (0.7974), $F_{\beta }^{\omega }$ (0.7363), $S_{\alpha }$ (0.8272), and $E_{\phi }^{m}$ (0.8883), while achieving the lowest error rates in *M* (0.0488) and $H_{\gamma }$ (659.3610). These results comprehensively validate both the independent effectiveness of our two core innovations and their powerful synergy when combined.

These results comprehensively validate our design choices and demonstrate that the powerful synergy between a well-designed fusion module and a selective feature injection strategy is the key to PMG-SAM’s good performance.

#### 4.7.3. Hyperparameter Sensitivity Analysis

**Figure 9:**
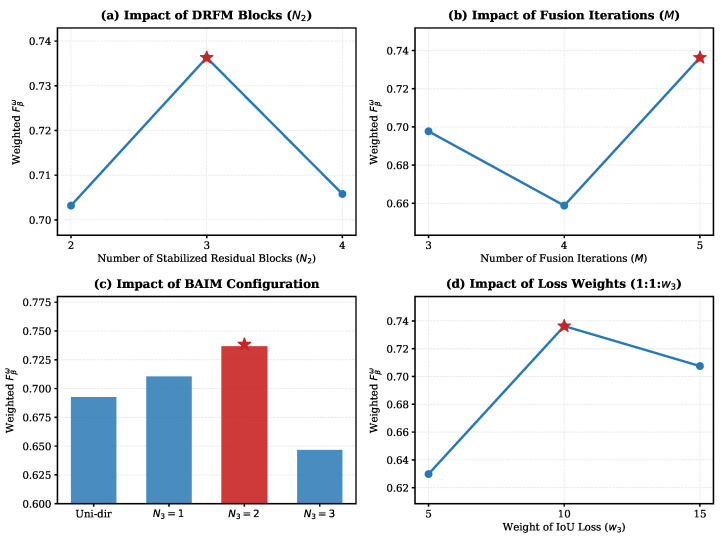
Sensitivity analysis of hyperparameters and structural components on the DIS-TE(1-4) dataset using Weighted F-measure ($F_{\beta }^{\omega }$). (**a**) Ablation on the number of Stabilized Residual Blocks ($N_{2}$). (**b**) Ablation on the number of residual fusion iterations (*M*). (**c**) Performance comparison between the unidirectional attention mechanism(Uni-dir) and the proposed Bidirectional Attention Interaction Module (BAIM) with varying module counts ($N_{3}$). (**d**) Analysis of the weight ratio for the IoU loss ($w_{3}$) within the total loss function. The red stars (⋆) and the red bar denote the selected optimal settings adopted in our final model.

To verify the rationality and robustness of the proposed framework, we conducted comprehensive sensitivity analyses on critical hyperparameters. All experiments were performed on the DIS-TE(1-4) dataset. The results are visualized in Figure 9. For detailed numerical results across all DIS5K datasets, please refer to Table A1 in Appendix A.

(1) Structure of DRFM ($N_{2}$ and *M*): As shown in Figure 9a,b, we analyzed the number of Stabilized Residual Blocks ($N_{2}$) and residual fusion iterations (*M*). The model achieves peak performance at $N_{2}=3$ and M=5. Reducing these parameters limits the network’s capacity to capture fine-grained details, while increasing them introduces redundancy without performance gains. Note that $N_{1}$ is fixed at 6 due to the inherent structure of the frozen backbones.

(2) Configuration of BAIM ($N_{3}$): We further investigated the optimal number of BAIM modules ($N_{3}$) and the necessity of the bidirectional mechanism. As illustrated in Figure 9c, the bidirectional configuration with $N_{3}=2$ significantly outperforms the unidirectional counterpart(Uni-dir) and other quantity settings ($N_{3}=1$ or 3). This confirms that two bidirectional interaction stages are sufficient to align multi-modal features effectively.

(3) Loss Function Weights: Finally, we evaluated the weight ratio of the loss function $L_{total}=w_{1}L_{bce}+w_{2}L_{dice}+w_{3}L_{iou}$. Since the IoU loss value is numerically smaller than BCE and Dice losses, a larger weight is typically required to balance the gradients. We tested different ratios for $w_{3}$ (5, 10, 15) while keeping $w_{1}=w_{2}=1$. Figure 9d demonstrates that the setting of 1:1:10 yields the best convergence and segmentation accuracy, validating our default configuration.

#### 4.7.4. Analysis of Prior Guidance Quality and Error Propagation

To validate the rationale behind our dual-branch encoder, we conducted a quantitative analysis of the intermediate Pre-Mask generated by the U^2^-Net branch.

Superiority of Structural Prior. We compared the Pre-Mask against semantic priors, specifically CLIPSeg [54], using the official weights and a generic prompt. As shown in Table 5, CLIPSeg yields an extremely low IoU of 0.024, indicating that semantic-based models struggle to capture the intricate boundary details required for DIS tasks. In contrast, our Pre-Mask achieves an IoU of 0.382 and an S-measure of 0.629, verifying that the U^2^-Net branch provides superior, shape-aware structural guidance.

**Figure 10:**
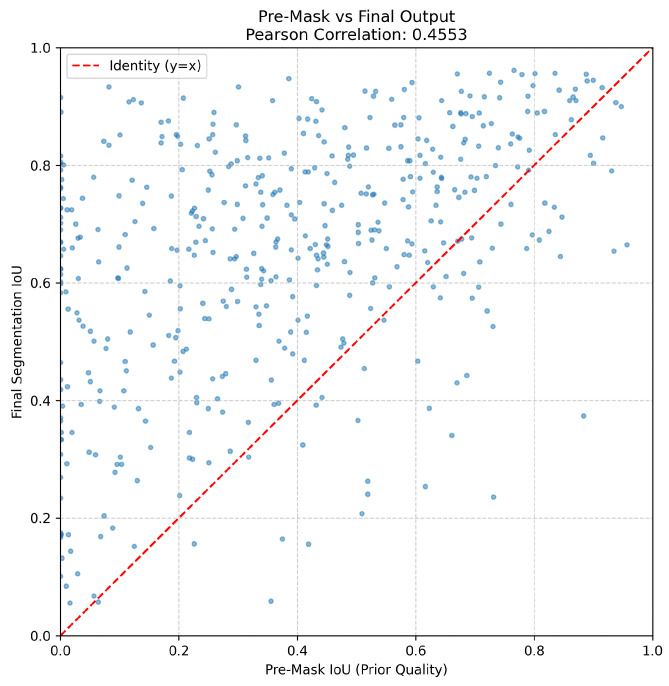
Error propagation analysis showing the correlation between Pre-Mask IoU and Final-Mask IoU on the DIS-VD dataset. The red dashed line represents the identity function (y=x). Points significantly above the line (especially in the low Pre-Mask IoU region) indicate the model’s robust capability to correct coarse priors and recover fine-grained details.

Refinement and Error Correction. Furthermore, our Final-Mask achieves a remarkable performance leap, boosting the IoU to 0.651 and reducing MAE to 0.047. To investigate the error propagation mechanism, we visualized the correlation between Pre-Mask and Final-Mask quality in Figure 10. The plot reveals a Pearson correlation of 0.455. Notably, a dense cluster of data points appears in the upper-left region (where Pre-Mask IoU <0.2 but Final-Mask IoU >0.6). This demonstrates a powerful error correction mechanism: even when the prior guidance is erroneous or fails to locate the target, the subsequent decoder effectively leverages image features to autonomously recover the correct segmentation, preventing error propagation.

## 5. Conclusions

In this paper, we presented PMG-SAM, a novel framework that represents a paradigm shift in automatic segmentation. Our work is motivated by the fundamental limitations of SAM’s “segment everything” mode: its localization blindness in complex scenes and the computational inefficiency of its grid-based prompting. To overcome these challenges, we proposed a new “locate-and-refine” architecture.

This new paradigm is operationalized by a Pre-Mask Generator, which performs the critical “locate” step. It leverages a synergistic dual-branch encoder and a novel Dense Residual Fusion Module (DRFM) to produce a high-quality, dense Pre-Mask that provides a strong global prior for the target. This internal guidance signal is then passed to an enhanced mask decoder, which executes the “refine” step, augmented by high-resolution features to ensure precise boundary delineation. This two-stage process replaces SAM’s exhaustive search with intelligent, targeted refinement, achieving both high accuracy and efficiency.

The effectiveness of this paradigm is empirically validated through extensive experiments on both salient and camouflaged targets. Our parameter-efficient PMG-SAM not only drastically outperforms the automatic modes of SAM and SAM2 but also surpasses their performance when provided with perfect ground-truth bounding box prompts. This key result highlights that our dense, internal guidance is a more powerful mechanism than sparse, external prompting. Furthermore, on the COS task, PMG-SAM demonstrates exceptional transfer learning and domain generalization capabilities, achieving good performance after minimal fine-tuning. Our ablation studies further confirmed that each component of our design is crucial to the framework’s success.

In summary, PMG-SAM and its underlying ‘locate-and-refine’ paradigm offer an effective and efficient solution to the inherent localization bottleneck of SAM’s automatic mode. Our work provides a valuable blueprint for developing future foundation models that are not only powerful and versatile but also truly and intelligently automatic in prompt-free scenarios.

## Figures and Tables

**Table 1:** Comparison of model complexity and inference efficiency. Metrics include Trainable Parameters (M), Total Parameters (M), FLOPs (G), Inference Speed (FPS), and Peak Memory (MB). All models were tested on a single NVIDIA Tesla A100 GPU with an input resolution of 1024×1024. Note that SAM and SAM2 variants were evaluated in their official automatic segmentation mode, which significantly impacts their speed due to the grid-based prompting strategy. The best results are highlighted in bold.

Method	Trainable Params (M)	Total Params (M)	FLOPs (G)	FPS	Peak Memory (MB)
SAM-B [1]	93.7	93.7	486.4	0.0072	2763.1
SAM-L [1]	312.0	312.0	1493.9	0.0073	4393.5
SAM-H [1]	641.0	641.0	2982.2	0.0070	5731.1
SAM2-T [40]	38.9	**38.9**	**103.0**	1.0179	**2366.0**
SAM2-B+ [40]	80.8	80.8	264.5	0.4647	2771.2
SAM2-L [40]	224.0	224.0	810.5	0.5347	3321.7
**PMG-SAM (Ours)**	**22.9**	136.0	1116.2	**4.8500**	3420.0

**Table 2:** **Quantitative comparisons of different methods.** In the ‘Mode’ column, ‘Auto’, ‘GT-Box’, and ‘Full-Sup’ are abbreviations for Automatic, GT-Bbox, and Fully supervised modes. The best results are highlighted in bold.

Methods	Mode	DIS-VD						DIS-TE1						DIS-TE2						DIS-TE3						DIS-TE4						DIS-TE(1-4)					
		$F_{\beta }^{\max}↑$	$F_{\beta }^{\omega }↑$	M↓	$S_{\alpha }↑$	$E_{\phi }^{m}↑$	$H_{\gamma }↓$	$F_{\beta }^{\max}↑$	$F_{\beta }^{\omega }↑$	M↓	$S_{\alpha }↑$	$E_{\phi }^{m}↑$	$H_{\gamma }↓$	$F_{\beta }^{\max}↑$	$F_{\beta }^{\omega }↑$	M↓	$S_{\alpha }↑$	$E_{\phi }^{m}↑$	$H_{\gamma }↓$	$F_{\beta }^{\max}↑$	$F_{\beta }^{\omega }↑$	M↓	$S_{\alpha }↑$	$E_{\phi }^{m}↑$	$H_{\gamma }↓$	$F_{\beta }^{\max}↑$	$F_{\beta }^{\omega }↑$	M↓	$S_{\alpha }↑$	$E_{\phi }^{m}↑$	$H_{\gamma }↓$	$F_{\beta }^{\max}↑$	$F_{\beta }^{\omega }↑$	M↓	$S_{\alpha }↑$	$E_{\phi }^{m}↑$	$H_{\gamma }↓$
SINetV2 [48]	Full-Sup	0.665	0.584	0.110	0.727	0.798	-	0.644	0.558	0.094	0.727	0.791	-	0.700	0.618	0.099	0.753	0.823	-	0.730	0.641	0.096	0.766	0.849	-	0.699	0.616	0.113	0.744	0.824	-	0.693	0.608	0.101	0.747	0.822	-
HRNet [46]	Full-Sup	0.726	0.641	0.095	0.767	0.824	-	0.668	0.579	0.088	0.742	0.797	-	0.747	0.664	0.087	0.784	0.840	-	0.784	0.700	0.080	0.805	0.869	-	0.772	0.687	0.092	0.792	0.854	-	0.743	0.658	0.087	0.781	0.840	-
STDC [47]	Full-Sup	0.696	0.613	0.103	0.740	0.817	-	0.648	0.562	0.090	0.723	0.798	-	0.720	0.636	0.092	0.759	0.834	-	0.745	0.662	0.090	0.771	0.855	-	0.731	0.652	0.102	0.762	0.841	-	0.710	0.628	0.094	0.754	0.832	-
IS-Net [2]	Full-Sup	**0.791**	0.717	0.074	0.813	0.856	1116	0.740	0.662	0.074	0.787	0.820	149	0.799	0.728	0.070	0.823	0.858	340	**0.830**	0.758	0.064	0.836	0.883	687	**0.827**	**0.753**	0.072	0.830	0.870	2888	**0.799**	0.726	0.070	0.819	0.858	1016
SAM-B [1]	Auto	0.215	0.132	0.258	0.398	0.392	1445	0.235	0.176	0.223	0.439	0.442	209	0.210	0.126	0.268	0.388	0.369	450	0.220	0.120	0.270	0.386	0.373	890	0.233	0.118	0.298	0.366	0.395	3624	0.224	0.135	0.265	0.395	0.395	1293
SAM-L [1]	Auto	0.278	0.231	0.325	0.401	0.462	1402	0.365	0.311	0.268	0.481	0.531	224	0.286	0.227	0.331	0.397	0.441	464	0.220	0.171	0.345	0.362	0.443	905	0.254	0.213	0.345	0.379	0.467	3528	0.281	0.230	0.322	0.404	0.471	1280
SAM-H [1]	Auto	0.283	0.241	0.344	0.395	0.475	1417	0.402	0.352	0.261	0.505	0.555	223	0.283	0.228	0.349	0.386	0.449	471	0.235	0.190	0.351	0.368	0.453	905	0.272	0.233	0.337	0.394	0.491	3502	0.298	0.251	0.325	0.413	0.487	1275
SAM2-T [40]	Auto	0.306	0.209	0.169	0.471	0.407	1417	0.352	0.253	0.142	0.506	0.450	189	0.311	0.204	0.168	0.468	0.394	443	0.308	0.203	0.169	0.470	0.391	877	0.268	0.179	0.192	0.445	0.382	3613	0.310	0.210	0.168	0.472	0.404	1280
SAM2-B+ [40]	Auto	0.428	0.311	0.156	0.515	0.477	1382	0.498	0.381	0.117	0.566	0.539	195	0.427	0.295	0.155	0.509	0.448	444	0.391	0.265	0.159	0.494	0.437	880	0.381	0.277	0.179	0.488	0.465	3509	0.424	0.305	0.153	0.514	0.472	1257
SAM2-L [40]	Auto	0.420	0.307	0.157	0.514	0.478	1385	0.494	0.382	0.117	0.570	0.550	196	0.442	0.310	0.147	0.518	0.464	444	0.390	0.266	0.157	0.497	0.437	877	0.385	0.279	0.177	0.491	0.464	3521	0.428	0.309	0.150	0.519	0.479	1259
SAM-B [1]	GT-Box	0.671	0.623	0.150	0.681	0.774	1554	0.747	0.703	0.105	0.754	0.829	286	0.687	0.635	0.143	0.692	0.784	590	0.624	0.573	0.171	0.647	0.745	1080	0.558	0.520	0.224	0.588	0.699	3667	0.654	0.608	0.161	0.670	0.764	1405
SAM-L [1]	GT-Box	0.739	0.698	0.117	0.739	0.817	1460	0.783	0.746	0.091	0.787	0.852	255	0.766	0.718	0.107	0.756	0.831	551	0.687	0.634	0.143	0.696	0.778	1021	0.613	0.576	0.191	0.639	0.734	3533	0.712	0.668	0.133	0.720	0.799	1340
SAM-H [1]	GT-Box	0.687	0.652	0.151	0.700	0.783	1468	0.755	0.721	0.106	0.766	0.833	244	0.708	0.666	0.141	0.713	0.791	543	0.629	0.583	0.176	0.654	0.748	997	0.576	0.545	0.218	0.611	0.707	3553	0.486	0.439	0.235	0.552	0.630	1325
SAM2-T [40]	GT-Box	0.739	0.702	0.107	0.748	0.830	1646	0.791	0.756	0.080	0.798	0.863	346	0.752	0.708	0.096	0.760	0.838	698	0.698	0.653	0.126	0.715	0.807	1203	0.622	0.587	0.179	0.652	0.748	3766	0.716	0.676	0.120	0.731	0.814	1503
SAM2-B+ [40]	GT-Box	0.765	**0.731**	0.104	0.766	0.840	1560	**0.834**	**0.805**	0.069	**0.829**	**0.888**	313	0.775	0.734	0.102	0.770	0.842	642	0.714	0.671	0.135	0.719	0.806	1169	0.633	0.601	0.188	0.657	0.741	3677	0.739	0.703	0.124	0.744	0.819	1450
SAM2-L [40]	GT-Box	0.743	0.707	0.107	0.752	0.819	1533	0.828	0.796	0.068	0.824	0.879	305	0.748	0.702	0.103	0.750	0.814	625	0.678	0.630	0.139	0.698	0.765	1127	0.603	0.569	0.187	0.639	0.719	3679	0.714	0.674	0.124	0.728	0.794	1434
SAM-H	Fine-tuned	0.378	0.316	0.167	0.503	0.798	907	0.325	0.267	0.130	0.508	0.825	176	0.364	0.300	0.166	0.498	0.798	365	0.369	0.304	0.181	0.489	0.785	663	0.383	0.324	0.192	0.488	0.778	2227	0.360	0.299	0.167	0.496	0.797	858
**PMG-SAM (Ours)**	Auto	**0.791**	0.726	**0.052**	**0.822**	**0.884**	**707**	0.768	0.688	**0.046**	0.810	0.858	**123**	**0.815**	**0.751**	**0.046**	**0.840**	**0.893**	**249**	0.826	**0.769**	**0.045**	**0.845**	**0.912**	**472**	0.783	0.738	**0.059**	**0.814**	**0.891**	**1793**	0.797	**0.736**	**0.049**	**0.827**	**0.888**	**659**

**Table 3:** **Quantitative comparisons of different methods on COD10K and NC4K.** Our method is evaluated in three settings: Zero-shot (trained on DIS5K only), Transfer Learning (fine-tuned on target dataset), and Domain Generalization (trained on one COS dataset, tested on another). **Bold**/Underline indicate the best/second-best results.

Methods	Mode	COD10K			NC4K		
		**AP**	**AP50**	**AP75**	**AP**	**AP50**	**AP75**
Mask R-CNN [49]	Fully supervised	28.7	**60.1**	25.7	36.1	**68.9**	33.5
PointSup [50]	Point-supervised	17.9	44.1	11.9	19.1	47.6	11.6
Tokencut [51]	Unsupervised	2.6	6.5	2.0	3.5	8.3	2.5
Cutler [52]	Unsupervised	11.7	29.1	7.3	15.5	37.9	10.5
TPNet [53]	Text-prompt	18.3	41.8	14.3	21.4	48.3	16.6
SAM-B [1]	Automatic	7.6	12.3	8.2	5.7	8.8	6.3
SAM-L [1]	Automatic	29.5	45.3	32.3	26.2	38.8	29.5
SAM-H [1]	Automatic	33.7	51.2	37.7	33.1	47.9	37.6
SAM2-T [40]	Automatic	3.1	4.0	3.4	3.4	4.2	3.8
SAM2-B+ [40]	Automatic	11.7	15.7	13.1	8.9	11.0	9.8
SAM2-L [40]	Automatic	10.6	13.2	12.1	8.8	10.3	9.6
SAM-H	Fine-tuned	**39.2**	58.3	**43.5**	37.6	53.5	41.5
PMG-SAM (Ours)	**Zero-shot**	12.9	31.2	8.8	21.4	45.5	18.0
PMG-SAM (Ours)	**Transfer Learning**	30.0	55.8	28.6	39.7	64.6	41.5
PMG-SAM (Ours)	**Domain Generalization**	29.9	53.6	29.3	**40.3**	66.5	**41.6**

**Table 4:** Ablation Study on Key Components of PMG-SAM. The best results are highlighted in bold.

Model Variant	DIS-TE(1-4)					
	$F_{\beta }^{\max}↑$	$F_{\beta }^{\omega }↑$	M↓	$S_{\alpha }↑$	$E_{\phi }^{m}↑$	$H_{\gamma }↓$
(a) Feature Fusion Module						
No Feature Fusion	0.7771	0.7029	0.0577	0.8135	0.8671	664.3980
Residual Fusion	0.7329	0.6271	0.0859	0.7648	0.8002	704.6665
(b) High-Resolution Features						
No High-Res Features	0.7584	0.6917	0.0595	0.8025	0.8661	772.5660
Only U^2^-Net	0.7409	0.6679	0.0667	0.7909	0.8476	764.1910
Both Backbones	0.7769	0.7014	0.0601	0.8071	0.8621	697.3505
**PMG-SAM (Ours)**	**0.7974**	**0.7363**	**0.0488**	**0.8272**	**0.8883**	**659.3610**

**Table 5:** Quantitative evaluation of the Pre-Mask quality compared to CLIP-based priors and the final output on the DIS-VD dataset. The generic prompt “a photo of a salient object” was used for CLIPSeg. The results demonstrate that our structural prior significantly outperforms semantic priors, and our full model achieves substantial refinement over the prior. The best results are highlighted in bold.

Method	IoU ↑	$F_{\beta }^{\max}↑$	MAE ↓	$S_{\alpha }↑$
CLIPSeg [54]	0.024	0.215	0.179	0.420
**Pre-Mask (Ours)**	0.382	0.515	0.117	0.629
**Final-Mask (Ours)**	**0.651**	**0.795**	**0.047**	**0.813**

**Figure A1:**
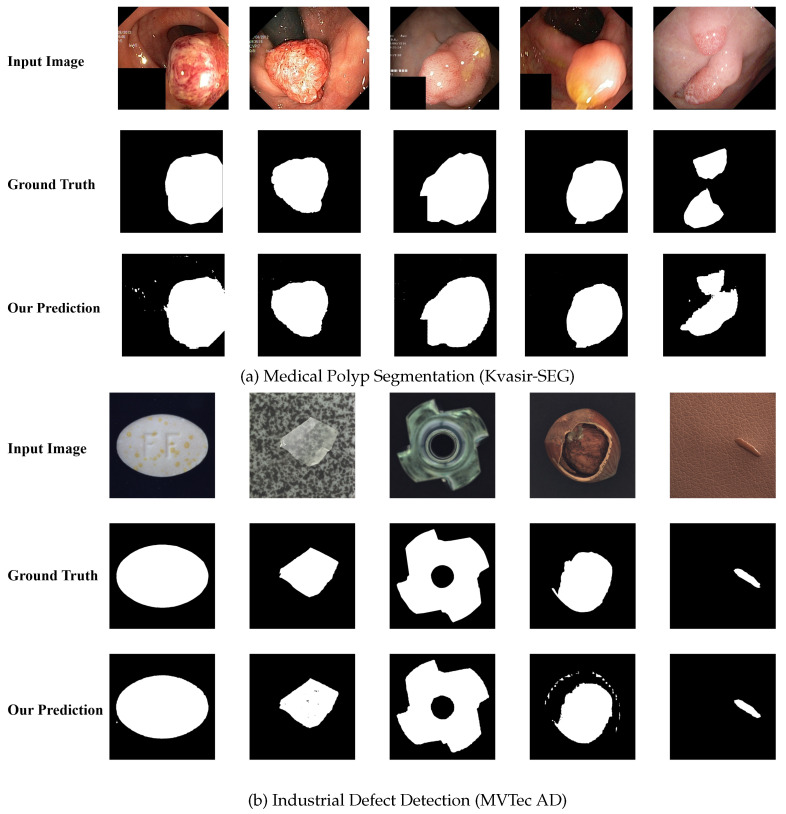
Qualitative visualization of zero-shot generalization on unseen domains.

## Data Availability

The experiments conducted in this research are based on the DIS5K, COD10K, and NC4K datasets, which are publicly available benchmark datasets. The DIS5K dataset can be accessed at https://xuebinqin.github.io/dis/index.html. The COD10K dataset can be accessed at https://drive.google.com/file/d/1YGa3v-MiXy-3MMJDkidLXPt0KQwygt-Z/view (for data) and https://drive.google.com/drive/folders/1Yvz63C8c7LOHFRgm06viUM9XupARRPif (for annotations). The NC4K dataset can be accessed at https://drive.google.com/file/d/1eK_oi-N4Rmo6IIxUNbYHBiNWuDDLGr_k/view (for data) and https://drive.google.com/drive/folders/1LyK7tl2QVZBFiNaWI_n0ZVa0QiwF2B8e (for annotations). All datasets were last accessed on 16 November 2025.

## Appendix A. Detailed Hyperparameter Sensitivity Results

**Table A1:** Detailed quantitative results of hyperparameter sensitivity analysis on all datasets and metrics. Lines containing ⋆ represent the default settings in this document.

Category	Setting	DIS-VD						DIS-TE1						DIS-TE2						DIS-TE3						DIS-TE4						DIS-TE(1-4)					
		$F_{\beta }^{\max}↑$	$F_{\beta }^{\omega }↑$	M↓	$S_{\alpha }↑$	$E_{\phi }^{m}↑$	$H_{\gamma }↓$	$F_{\beta }^{\max}↑$	$F_{\beta }^{\omega }↑$	M↓	$S_{\alpha }↑$	$E_{\phi }^{m}↑$	$H_{\gamma }↓$	$F_{\beta }^{\max}↑$	$F_{\beta }^{\omega }↑$	M↓	$S_{\alpha }↑$	$E_{\phi }^{m}↑$	$H_{\gamma }↓$	$F_{\beta }^{\max}↑$	$F_{\beta }^{\omega }↑$	M↓	$S_{\alpha }↑$	$E_{\phi }^{m}↑$	$H_{\gamma }↓$	$F_{\beta }^{\max}↑$	$F_{\beta }^{\omega }↑$	M↓	$S_{\alpha }↑$	$E_{\phi }^{m}↑$	$H_{\gamma }↓$	$F_{\beta }^{\max}↑$	$F_{\beta }^{\omega }↑$	M↓	$S_{\alpha }↑$	$E_{\phi }^{m}↑$	$H_{\gamma }↓$
DRFM Blocks ($N_{2}$)	$N_{2}=2$	0.7598	0.6934	0.0596	0.8069	0.8644	717	0.7293	0.6368	0.0611	0.7835	0.8248	129	0.79	0.7196	0.0553	0.8245	0.8737	262	0.8033	0.741	0.0508	0.8307	0.8984	483	0.7669	0.7154	0.0647	0.8031	0.8822	1806	0.7711	0.7032	0.058	0.8104	0.8698	670
	$⋆N_{2}=3$	0.7905	0.7262	0.0519	0.8218	0.8841	707	0.7679	0.6875	0.0456	0.8096	0.8576	123	0.8147	0.7509	0.0459	0.8402	0.8928	249	0.8261	0.7689	0.0448	0.8451	0.9116	472	0.7831	0.7382	0.0591	0.8141	0.8913	1793	0.7974	0.7363	0.0488	0.8272	0.8883	659
	$N_{2}=4$	0.7611	0.6946	0.0599	0.8065	0.8656	731	0.7345	0.6438	0.0583	0.7877	0.8288	127	0.7913	0.7247	0.0532	0.8276	0.8763	261	0.8017	0.7384	0.0505	0.8315	0.8968	490	0.766	0.7165	0.0655	0.8038	0.8779	1835	0.7727	0.7058	0.0569	0.8127	0.87	678
DRFM Iterations (*M*)	M=3	0.7663	0.6921	0.0611	0.811	0.8613	735	0.7315	0.624	0.062	0.7842	0.8159	135	0.7846	0.7133	0.0573	0.8267	0.8678	267	0.8038	0.7379	0.0523	0.836	0.8944	497	0.7685	0.7155	0.0652	0.809	0.8792	1845	0.7712	0.6977	0.0592	0.814	0.8643	686
	M=4	0.7478	0.6454	0.0772	0.7782	0.8237	740	0.7026	0.5774	0.081	0.7501	0.776	132	0.7736	0.6775	0.0706	0.8006	0.8402	264	0.7914	0.6698	0.0637	0.8098	0.8863	498	0.7556	0.6806	0.0805	0.7834	0.8475	1865	0.7557	0.6588	0.0739	0.786	0.8325	690
	⋆M=5	0.7905	0.7262	0.0519	0.8218	0.8841	707	0.7679	0.6875	0.0456	0.8096	0.8576	123	0.8147	0.7509	0.0459	0.8402	0.8928	249	0.8261	0.7689	0.0448	0.8451	0.9116	472	0.7831	0.7382	0.0591	0.8141	0.8913	1793	0.7974	0.7363	0.0488	0.8272	0.8883	659
BAIM Configuration	Uni-dir	0.7444	0.682	0.0672	0.7958	0.8539	726	0.7195	0.6304	0.0639	0.7774	0.8178	130	0.7743	0.7075	0.0609	0.8136	0.8636	266	0.7889	0.7271	0.0558	0.8211	0.8889	492	0.7542	0.7053	0.0699	0.7933	0.8719	1846	0.7582	0.6926	0.0626	0.8013	0.8605	683
	$N_{3}=1$	0.763	0.7017	0.0568	0.8115	0.8738	713	0.7336	0.6458	0.0567	0.7875	0.8312	128	0.7953	0.7311	0.0508	0.8316	0.8833	257	0.8061	0.7462	0.0487	0.8342	0.9056	481	0.7671	0.7186	0.0639	0.8048	0.8819	1794	0.7749	0.7104	0.055	0.8145	0.8755	665
	$⋆N_{3}=2$	0.7905	0.7262	0.0519	0.8218	0.8841	707	0.7679	0.6875	0.0456	0.8096	0.8576	123	0.8147	0.7509	0.0459	0.8402	0.8928	249	0.8261	0.7689	0.0448	0.8451	0.9116	472	0.7831	0.7382	0.0591	0.8141	0.8913	1793	0.7974	0.7363	0.0488	0.8272	0.8883	659
	$N_{3}=3$	0.7424	0.6378	0.0797	0.7735	0.8142	739	0.6857	0.5611	0.0862	0.7416	0.759	131	0.7678	0.6665	0.0747	0.7935	0.8307	265	0.7818	0.6856	0.0688	0.8008	0.851	497	0.7509	0.6735	0.0832	0.779	0.8407	1893	0.7465	0.6467	0.0782	0.7787	0.8203	696
Loss Weights ($w_{3}$)	$w_{3}=5$	0.7362	0.6188	0.0899	0.7636	0.7979	747	0.687	0.5367	0.096	0.7291	0.7376	134	0.7654	0.6473	0.085	0.7835	0.8136	265	0.7861	0.6729	0.0749	0.7977	0.8417	505	0.7562	0.6621	0.0875	0.7788	0.8337	1893	0.7467	0.6298	0.0859	0.7723	0.8067	699
	$⋆w_{3}=10$	0.7905	0.7262	0.0519	0.8218	0.8841	707	0.7679	0.6875	0.0456	0.8096	0.8576	123	0.8147	0.7509	0.0459	0.8402	0.8928	249	0.8261	0.7689	0.0448	0.8451	0.9116	472	0.7831	0.7382	0.0591	0.8141	0.8913	1793	0.7974	0.7363	0.0488	0.8272	0.8883	659
	$w_{3}=15$	0.7672	0.6954	0.0615	0.8021	0.8594	721	0.7349	0.6457	0.06	0.7827	0.8239	126	0.7915	0.7185	0.0572	0.8194	0.868	258	0.809	0.7415	0.0516	0.8279	0.8939	481	0.7798	0.7245	0.0631	0.8044	0.8826	1797	0.7788	0.7075	0.058	0.8086	0.8671	666

## Appendix B. Qualitative Analysis on Out-of-Distribution Domains

To respond to the need for validating the generalization capability of the proposed model across a broader set of tasks, we conducted zero-shot inference on two distinct out-of-distribution datasets: medical imaging and industrial defect detection. It is important to note that our model was trained exclusively on the DIS dataset and was not fine-tuned on any medical or industrial data.

The PMG-SAM model, trained solely on the DIS dataset, is directly applied to (a) the Kvasir-SEG dataset [55] for polyp segmentation and (b) the MVTec AD dataset [56] for industrial defect detection. In (a), the model accurately delineates the polyp boundaries despite low contrast and blurred edges. In (b), it successfully identifies diverse defects and separates the target from complex textures. These results demonstrate that the proposed Pre-Mask mechanism learns a robust, class-agnostic representation of “objectness” that generalizes effectively to diverse downstream tasks without specific adaptation.
